# Purification and characterization of an amyloglucosidase from an ericoid mycorrhizal fungus (*Leohumicola incrustata*)

**DOI:** 10.1186/s13568-018-0685-1

**Published:** 2018-09-29

**Authors:** O. R. Adeoyo, B. I. Pletschke, J. F. Dames

**Affiliations:** 1 Department of Biochemistry and Microbiology, Rhodes University, P.O. Box 94, Grahamstown, 6140 South Africa; 2 Department of Microbiology, Adekunle Ajasin University, P.M.B. 001, Akungba-Akoko, Ondo State Nigeria

**Keywords:** Amyloglucosidase, Chromatography, Ericoid mycorrhiza, Starch, Zymography

## Abstract

This study aimed to purify and characterize amyloglucosidase (AMG) from *Leohumicola incrustata.* AMG was purified to homogeneity from cell-free culture filtrate of an ERM fungus grown in a modified Melin–Norkrans liquid medium. The molecular mass of the AMG was estimated to be 101 kDa by combining the results of Sephadex G-100 gel filtration, sodium dodecyl sulphate–polyacrylamide gel electrophoresis, and zymography. The *K*_*m*_ and *k*_*cat*_ values were 0.38 mg mL^−1^ and 70 s^−1^, respectively, using soluble starch as a substrate. The enzyme was stable at 45 °C (pH 5.0), retaining over 65% activity after a pre-incubation period of 24 h. The metal inhibition profile of the AMG showed that Mn^2+^ and Ca^2+^ enhanced activity, while it was stable to metals ions, except a few (Al^3+^, Co^2+^, Hg^2+^ and Cd^2+^) that were inhibitory at a concentration higher than 5 mM. Thin layer chromatography revealed that only glucose was produced as the product of starch hydrolysis. The amylase from *L. incrustata* is a glucoamylase with promising characteristics such as temperature stability over an extended period, high substrate affinity and stability to a range of chemicals. Also, this study reports for the first time the possibility of using some culturable ERM fungi to produce enzymes for the bio-economy.

## Introduction

*Leohumocola incrustata* is a genus of mycorrhizas commonly found within the roots of ericaceous plants. Ericaceous plants belong to a family of plants that can alter their physiological or morphological characteristics in nutrient deficient ecosystems. The survival of ericaceous plants in nutrient–deficient soils depends strongly on the symbiotic association that exists between them and mycorrhizal fungi, where nutrients are made available from soil organic matter through the assistance of hydrolytic enzymes. Ericoid mycorrhizal (ERM) fungi have been found to produce hydrolytic enzymes (Cairney and Burke 1994, 1998) which include the production of amyloglucosidase. Other reports have also indicated that the enzymatic degradation of organic polymers in the soil and the transfer of some of the resulting products to the root is a significant benefit to the growth and development of ericaceous plants (Smith and Read 2008). A new area of biotechnological research now focuses on the production and characterization of enzymes from various sources with unique properties (Karim et al. 2017). Some of the desired characteristics of industrial enzymes include thermostability, specificity and pH stability. The application of amylases has increased over the years in areas such as juice processing, processing of starch, resizing of textiles, paper sizing, detergent additives, malting barley and bakery industries (Singh et al. 2016; Rana et al. 2017; Raveendran et al. 2018).

Alpha-amylase and glucoamylase are the two major classes of amylases, mostly identified among microorganisms (John 2017). Alpha-amylases are exo-acting enzymes that randomly cleave the 1,4-α-d-glucosidic linkages between adjacent glucose units in the linear amylose chain. Amyloglucosidase (AMG, glucoamylase, EC 3.2.1.3) is an enzyme that is capable of hydrolyzing the α-1,4 glycosidic bonds from the non-reducing ends of starch to produce glucose. It is also an exo-acting enzyme that catalyzes the production of β-d-glucose from the non-reducing ends of substrates that include starch, and maltooligosaccharides by consecutively hydrolyzing α-1,4 and α-1,6 linkages (Sauer et al. 2000; Riaz et al. 2012; John 2017; Raveendran et al. 2018).

Starch is one of the most abundantly distributed polysaccharides produced by plants. It is made up of two molecular weight polymers, amylose and amylopectin. Amylose is a linear chain of glucose residues linked by α-1,4 bonds while amylopectin is a branched polymer with α-1,4-linked and α-1,6-linked glucose residues. AMG serves as a raw material for fermentation in the production of ethanol, glucose syrups, and in some cases can be used to improve barley mash for beer production (Aiyer 2005; Zambare 2010). Consequently, AMG can be considered as an economically significant enzyme because of its effectiveness in hydrolyzing starch and some oligosaccharides into β-d-glucose. Glucoamylase has been reported in *Rhizopus oryzae* (Morita and Fujio, 2000), *Aspergillus awamori* (Negi and Banerjee 2009), *Aspergillus niger* (Slivinski et al. 2011), as well as in two ectomycorrhizal fungi, *Tricholoma matsutake* and *Lyophyllum shimeji* (Hur et al. 2001; Kusuda et al. 2004). Kusuda et al. 2004 characterized an extracellular glucoamylase from *L. shimeji*, stating that the enzyme was most active at around 40 °C. More recently, this enzyme has been reported to be produced from some microorganisms, viz, *Aspergillus flavus* (Karim et al. 2017) and *Tetracladium* sp. (Carrasco et al. 2017). In this study, an amyloglucosidase from *L. incrustata* was purified and characterized.

## Materials and methods

### Culture

An ericoid mycorrhizal fungus, *L. incrustata* (Isolate code ChemRU330/Genbank accession no: MF374380/South African National Collection of Fungi accession no: PPRI 17268), was obtained from the Mycorrhizal Research Laboratory, Rhodes University, Department of Biochemistry and Microbiology, Rhodes University, Grahamstown, South Africa. The isolate was maintained on potato dextrose agar (PDA). Incubation was performed at 28 °C for 21 days. The mycelia were kept on PDA, stored at 4 °C and regularly subcultured throughout the study period.

### Production of amylase

Soluble Starch (1% w/v, Merck, Cas # 9005-84-9) was added to a 1000 mL^−1^ Erlenmeyer flask in an modified Melin–Norkrans (MMN) broth with the composition: (g L^−1^): glucose 1.0; yeast extract 3.0; malt extract 1.0; (NH_4_)_2_HPO_4_ 0.25; KH_2_PO_4_ 0.50; MgSO_4_·7H_2_O 0.15; CaCl_2_ 0.05; NaCl 0.025; thiamine-HCl 100 µg L^−1^, ZnSO_4_·7H_2_O 0.003, and 1.2 mL of FeCl_3_ (1% w/v). Production medium was thoroughly mixed, and a 250 mL was placed in a 500 mL shake flask. The medium was sterilized at 121 °C for 15 min and was inoculated with five discs of 5 mm mycelial plugs of the fungus. The uninoculated medium was used as a control. Growth was allowed to proceed at 28 °C in the dark for 3 weeks on a rotary incubator shaker at 150 rpm. After 42 days of growth, the cultures were homogenised using IKA’s ULTRA-TURRAX homogeniser (20,000 rpm), and centrifugation was performed at 10,000×*g* for 15 min to obtain crude enzyme filtrates (supernatant) using a Beckman Coulter Avanti-J high-speed centrifuge.

### Assay for amylase

A dinitrosalicylic acid (DNS) assay (Miller 1959) was conducted by adding 1% (w/v) soluble starch to a volume of 10 mL sodium acetate buffer (pH 5.0) in a Schott bottle and boiled for 30 s. A volume of 100 μL of crude enzyme and uninoculated control was added to 300 μL of the soluble starch-containing medium in triplicate, while the blank contained 400 μL buffer. All samples were incubated at 37 °C for 1 h, followed by centrifugation at 6000×*g* for 2 min. A 300 μL aliquot of DNS was added to 150 μL of each supernatant sample. This was followed by boiling on a heating block at 100 °C for 5 min after which it was cooled on ice for 5 min. A volume of 250 μL of each sample was placed into each well of a 96-well plate and read with the aid of a spectrophotometer (BioTek’s Synergy Mx) at a wavelength of 540 nm. The supernatant was taken to determine the reducing sugar using DNS assay with glucose as a standard.

### Protein analysis

Protein content was determined with Bradford reagent assay kit using bovine serum albumin as standard (Bradford 1976).

### Ammonium sulphate precipitation

A 120 mL volume of the crude enzyme was brought to 80% saturation with solid ammonium sulphate according to the method of Kusuda et al. (2004). The mixture was left overnight at 4 °C on a magnetic stirrer. The pellet obtained from the centrifuged mixture was re-dissolved in 20 mM sodium acetate buffer (pH 5.0) to make up a volume of 10 mL.

### Dialysis of the partially purified enzyme

A pre-treated dialysis bag was used for the dialysis (10 kDa cut-off) of the enzyme collected after ammonium sulphate precipitation. The partially purified enzyme (10 mL) was dialyzed against 20 mM sodium acetate buffer (pH 5.0) at 4 °C with three changes of a buffer according to the method described by Kusuda et al. (2004).

### Gel filtration

A vertical glass chromatographic column (1.5 × 50 cm) was packed using Sephadex G-100. The dialyzed enzyme solution (1.5 mL) was used after it was concentrated by ultrafiltration (Amicon Ultra-100 centrifugal filter device; cut-off 10 kDa). Gel filtration chromatography was carried out using sodium acetate buffer (20 mM, pH 5.0), at a flow rate of 1.2 mL min^−1^. All fractions collected were subjected to analysis by measuring the absorbance at 280 nm, followed by the activity assay, the active fractions (fractions no. 36–40; 5 mL) were pooled together afterwards.

### Identification of hydrolytic products using thin layer chromatography (TLC)

Soluble starch was used as the substrate. The mixture was incubated in a dry heating block at 45 °C for 1 min up to 24 h and samples were removed at various time intervals for analysis. Samples were then boiled for 5 min at 100 °C to denature the proteins and then centrifuged at 6000×*g* for 5 min. The supernatant (80 µL) was used for TLC analysis. Identical volumes (5 µL) of the supernatant were applied to Silica Gel 60G F254 HPTLC plates (Merck, Darmstadt, Germany). Plates were developed twice with n-butanol: acetic acid: water (2:1:1, v/v/v). Then, to detect carbohydrates, plates were briefly submerged in methanol containing 5% (v/v) sulfuric acid and 0.3% α-naphthol. Plates were then air dried, heated at 120 °C for 10 min and observed for the bands formed by various hydrolytic products.

### AMG activity staining (zymography)

The enzyme from crude extract was analyzed by its native protein pattern using the copolymerization method of Martinez et al. (2000). Sample was diluted in 1:1 in sample buffer (0.125 M Tris; 20% v/v glycerol; 0.04% v/v bromophenol blue) and boiled at 95 °C for 5 min. The resolving gel (12%) consisted of:- polyacrylamide 4.0 mL, 1.5 M Tris–HCl buffer (pH 8.8) 2.5 mL, distilled water 2.25 mL, 2% w/v soluble starch 1.25 mL, 10% w/v sodium dodecyl sulfate (SDS) 100 µL, 10% w/v ammonium persulfate 50 µL, and *N*,N,*N′*,*N′*-tetramethylethylenediamine (TEMED) 5 µL. The stacking gel (4%) consisted of: polyacrylamide 0.65 mL, 0.5 M Tris–HCl buffer (pH 6.8) 1.25 mL, distilled water 3.05 mL, 10% w/v SDS 50 µL, 10% w/v ammonium persulfate 25 µL, and TEMED 5 µL. Starch-SDS-PAGE (PAGE—polyacrylamide gel electrophoresis) was carried out in two phases viz. gels were first subjected to a constant voltage of 30 V for 30 min to ensure the tracking dye (bromophenol blue) enters the separating gel. After this, gels were finally subjected to a constant voltage of 100 V for 75 min. Electrophoresis was carried at a temperature of 0–2 °C to allow the enzyme to migrate inactively without hydrolyzing the starch. After electrophoresis, the gel was washed in distilled water and then incubated in 0.1 M phosphate-citrate and 0.05 M NaCl buffer (pH 6.0) for 3 h at 39 °C. Again, gels were washed and fixed in 12% trichloroacetic acid (TCA) for 10 min, followed by another washing and gently shaking (50 rpm) in 2.5% w/v Triton X-100 for 1 h at 4 °C to remove SDS and restore activity. Finally, the gels were stained with Lugol solution (6.7 mg mL^−1^ KI and 3.3 mg mL^−1^ I_2_), and photographs were taken.

### Molecular weight determination by SDS-PAGE electrophoresis

SDS-PAGE was performed using 8 × 10 × 0.75 cm gels in a Mini-Protean II (Bio-Rad) gel apparatus. Samples were treated with reducing (containing 2-mercaptoethanol) sample buffer and boiled for 5 min before loading the gel. After electrophoresis, proteins in the gel were visualized by staining with Coomassie Blue R-250 (Laemmli 1970).

### Effect of pH on activity and stability of the AMG

The optimum pH for the enzyme was determined by incubating enzyme with the substrate (1%, w/v) prepared in 0.1 M buffer having pH values of 1.0, 2.0 (hydrochloric acid–potassium chloride); 3.0 (citrate); 4.0, 5.0 (acetate); 6.0, 7.0 (phosphate); 8.0 and 9.0 (Tris–HCl) at a temperature of 45 °C for 1 h, after which the enzyme activity was measured. The pH stability was determined by pre-incubating the enzyme in buffers of different pH (3.0, 4.0, 5.0, 6.0, 7.0 and 8.0) at 37 °C for 6, 12, 18, 24 and 30 h before determining the residual activity by the standard procedure.

### Effect of temperature on activity and stability of the AMG

The optimum pH for the enzyme was determined by incubating enzyme with the substrate (1% w/v) prepared in 0.1 acetate buffer (pH 5.0). The enzyme was incubated at different temperatures of 4, 20, 30, 40, 50, 60, 70 and 80 °C for 1 h, after which, the enzyme activity was measured. The temperature stability was investigated by pre-incubating the enzyme at different temperatures (37, 45, 50, 60, and 70 °C) for 6, 12, 18, 24 and 30 min before determination of the residual activity by the standard procedure.

### Effect of metal ions and other additives on the activity of the AMG

Metal ions such as K^+^, Na^+^, Ca^2+^, Mn^2+^, Fe^2+^, Mg^2+^, Al^3+^, Hg^2+^, and Cd^2+^ (all supplied in chloride form) were each applied to determine the effect on the activity of the enzyme. Each metal ion was used at a concentration of 1, 5, and 10 mM, while incubation was performed at 45 °C for 1 h. The following compounds—sodium azide (NaN_3_), dimethyl sulfoxide (DMSO), ethylenediaminetetraacetic acid (EDTA), Tween 20, SDS, and indole-3-acetic acid (IAA) were tested for their inhibitory effect on AMG activity. The purified enzyme was incubated at 45 °C for 1 h in 20 mM sodium acetate buffer (pH 5.0), containing each inhibitor or denaturing agent at final concentrations of 1, 5, and 10 mM, respectively. The relative enzyme activity was determined under standard assay conditions.

### Enzyme kinetics

The Michaelis–Menten substrate saturation curve was used to determine the *K*_*m*_ and *V*_*max*_ value of the AMG by measuring the rate of soluble hydrolysis under standard assay conditions. The reaction mixture was 20 mM acetate buffer (pH 5.0), with the soluble starch substrate at concentrations ranging from 0.2 to 10 mg mL^−1^. The values for *K*_*m*_ and *V*_*max*_ were then determined using KaleidaGraph.

### Statistical analysis

All experiments were carried out in triplicate. Data were analysed on Microsoft^®^ Excel software, and all values were presented as the standard errors of the means (± SEM).

## Results

### Purification of enzyme

**Fig. 1:**
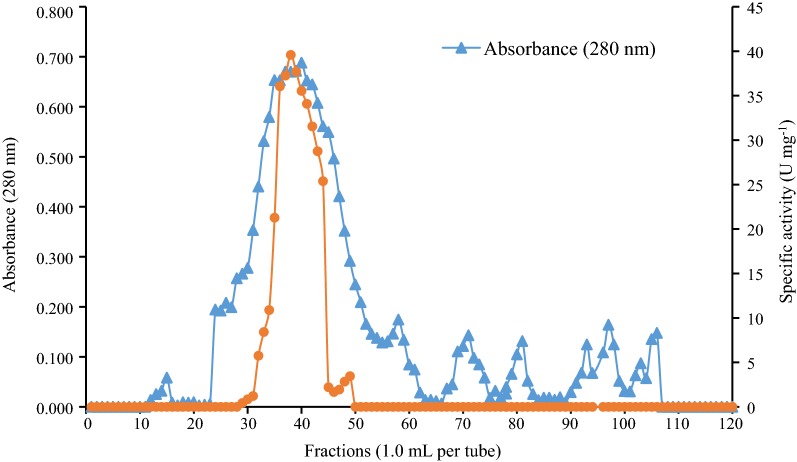
Chromatogram of the crude enzyme fractions of *Leohumicola incrustata* on a Sephadex G-100 chromatographic column (flow rate: 1.2 mL min^−1^)

The production of AMG from *L. incrustata* was induced by the addition of soluble starch into a mineral medium at a temperature of 28 °C for 42 days (pH 5.0). Table 1 summarizes the purification steps of the AMG solution prepared from the cell-free culture filtrate (120 mL). Partial purification of the AMG by ammonium sulphate [(NH_4_)_2_SO_4_] precipitation was the first step of the purification procedure; the filtrate was precipitated at 80% saturation. The ammonium sulphate fraction yielded 71% activity with purification fold of 13.7 and specific activity of 15.8 U mg^−1^ protein. The dialysis step produced a 54% activity with purification fold of 18.61 and specific activity of 21.5 U mg^−1^ protein. The elution profile on Sephadex G-100 showed that peak (b) (Fig. 1) exhibited high AMG activity, while other peaks did not show any noticeable amylase activity. The gel purification step yielded 27% activity with purification fold of 23.5 and a specific activity of 27.17 U mg^−1^ protein (Table 1).

**Table 1:** Purification table of AMG from *Leohumicola incrustata*

Purification step	Total protein (mg)	Total activity (U)	Specific activity (U mg^−1^ protein)	Purification fold	Yield (%)
Cell-free filtrate	234.709	271.080	1.155	1.000	100
(NH_4_)_2_SO_4_	12.152	192.139	15.812	13.690	71
Dialysis	6.848	147.207	21.495	18.611	54
Gel filtration	2.698	73.326	27.174	23.528	27

### Enzyme characterization

**Fig. 2:**
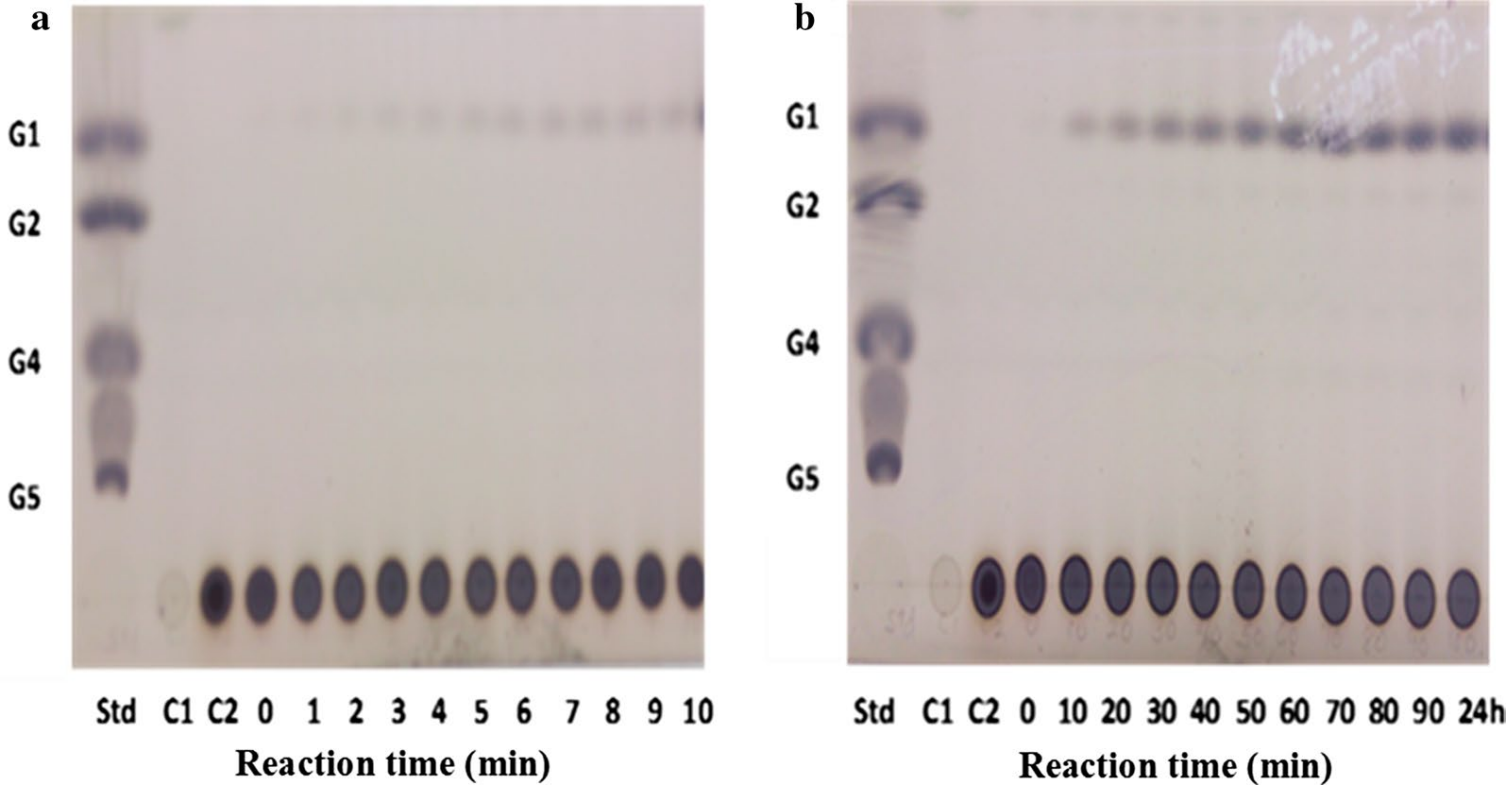
Thin-layer chromatograms of hydrolyzates. Plates were developed twice with *n*-butanol: acetic acid: water (2:1:1, v/v/v). The plates were briefly submerged in methanol containing 5% (v/v) sulfuric acid and 0.3% α-naphthol. Standard (Std): glucose (G1), maltose (G2), maltotetraose (G4), and maltopentaose (G5), substrate control (C1), enzyme control (C2), reaction time: 0–90 min and 24 h

**Fig. 3:**
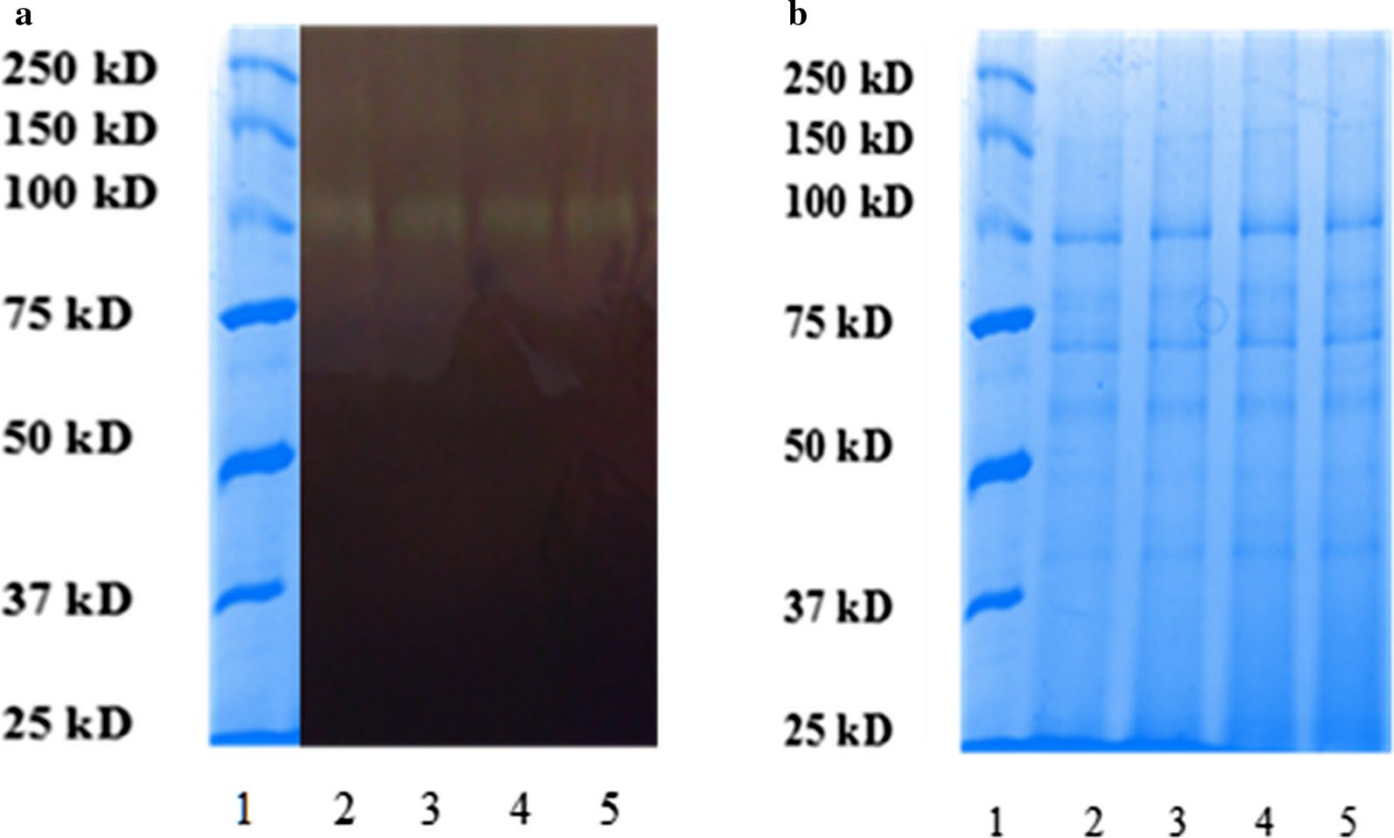
12% Zymogram and SDS-PAGE analysis of the AMG prepared according to the method described by Martinez et al. (2000). **a** Lane 1 is precision plus unstained protein standard (BIO-RAD), lanes 2–5 is a crude enzyme of *Leohumicola incrustata* (iodine staining); **b** lane 1 is precision plus unstained protein standard (BIO-RAD), lanes 2–5 is SDS-PAGE of crude AMG

**Fig. 4:**
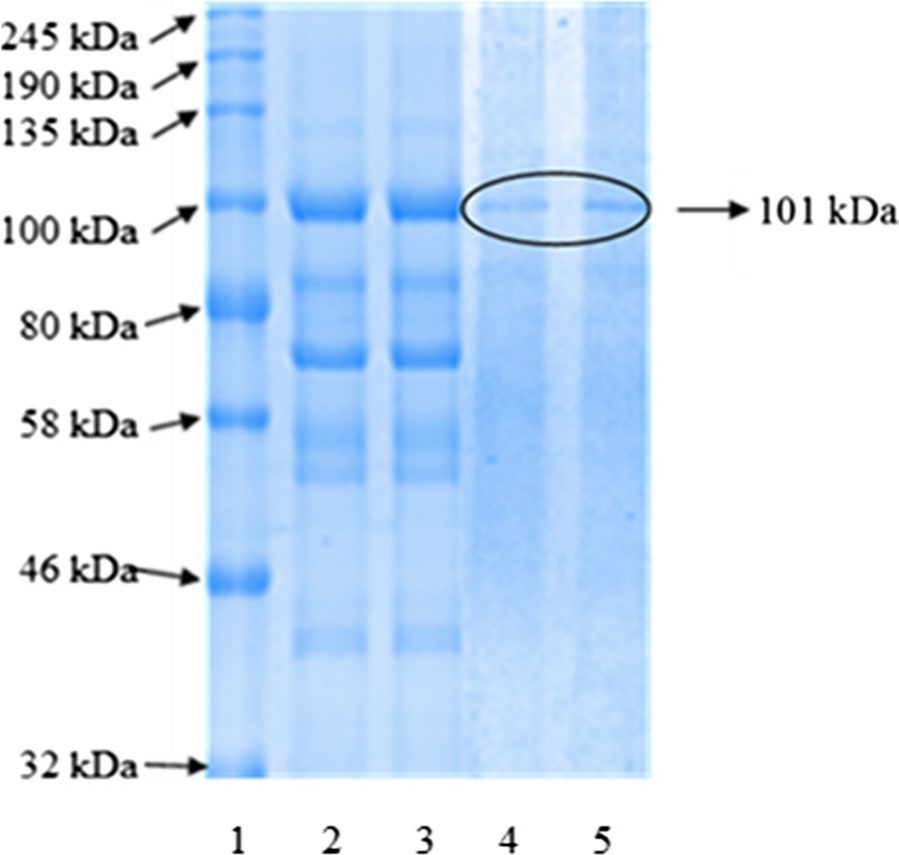
12% SDS-PAGE analysis of the AMG (Laemmli 1970): lane 1 is colour pre-stained standard, broad range (BioLabs); AMG crude enzyme (lanes 2, 3); the post-gel filtration chromatography protein bands (lanes 4, 5)

Fractions showing the highest AMG activities (fractions 36–40) were pooled for characterization studies (Fig. 1). Figure 2 shows the pattern of TLC of AMG hydrolysis from *L. incrustata*. The reaction time ranged from 1 to 10 min (Fig. 2a), 10 min to 90 min and 24 h (Fig. 2b) at 45 °C. A single band of clearance zone was revealed after activity gel analysis, which indicated that the *L. incrustata* released a single isoform of the AMG (Fig. 3). Also, SDS-PAGE was carried out to confirm homogeneity of the enzyme using a 12% gel. Thus Fig. 3 shows the results of SDS-PAGE and zymography. The molecular weight of the enzyme was estimated to be 101 kDa using a standard curve of log (MW) versus R_f_. Figure 4 shows the result of gel filtration on SDS-PAGE to obtain a single homogeneous protein band (101 kDa).

### Effect of pH on enzyme activity and stability

**Fig. 5:**
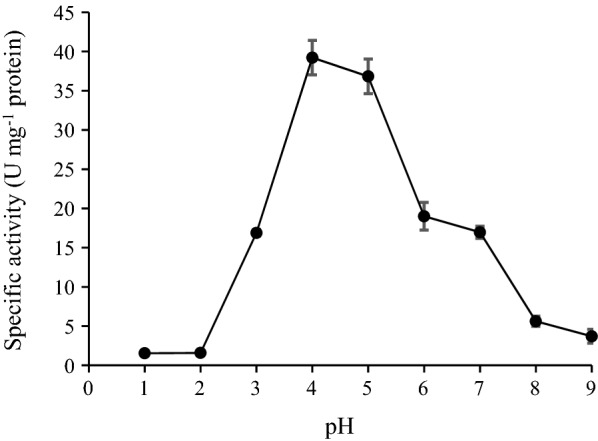
Effect of pH on *Leohumocola incrustata* specific activity. The enzyme was incubated at 45 °C for 1 h. All error bars are represented as the standard errors of the means (SEM)

**Fig. 6:**
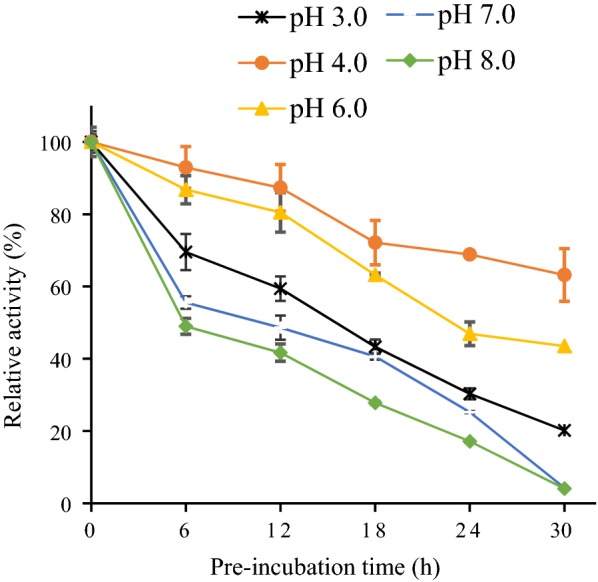
Effect of pH on stability of the AMG. The enzyme was pre-incubated (37 °C) for 6, 12, 18, 24, and 30 h before the assay was carried out at 45 °C for 1 h. All error bars are represented as the standard errors of the means (SEM)

Effect of pH on enzyme activity was tested using reaction mixtures buffered at different pH values ranging from 1.0 to 9.0, for optimal activity (Fig. 5). The findings showed that the highest activity of the purified enzyme was obtained at pH 4.0, and more than 50% activity was retained between pH 4.0 to 6.0. At pH 5.0 (37 °C) over a period of 24 h, the enzyme was 100% stable (Fig. 6). Hence, the enzyme displayed optimal activity under a mildly acidic pH. The AMG activity was also observed to be stable (above 50%) up to 6 h at a pH of 7.0 (37 °C) but dropped rapidly after 12 h (Fig. 6).

### Effect of temperature on activity and stability of AMG

**Fig. 7:**
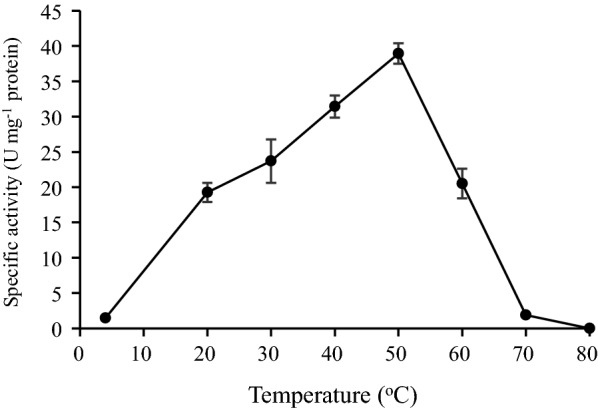
Effect of temperature on the AMG activity. The enzyme was incubated at the temperature of 4 to 80 °C for 1 h. All error bars are represented as the standard errors of the means (SEM)

**Fig. 8:**
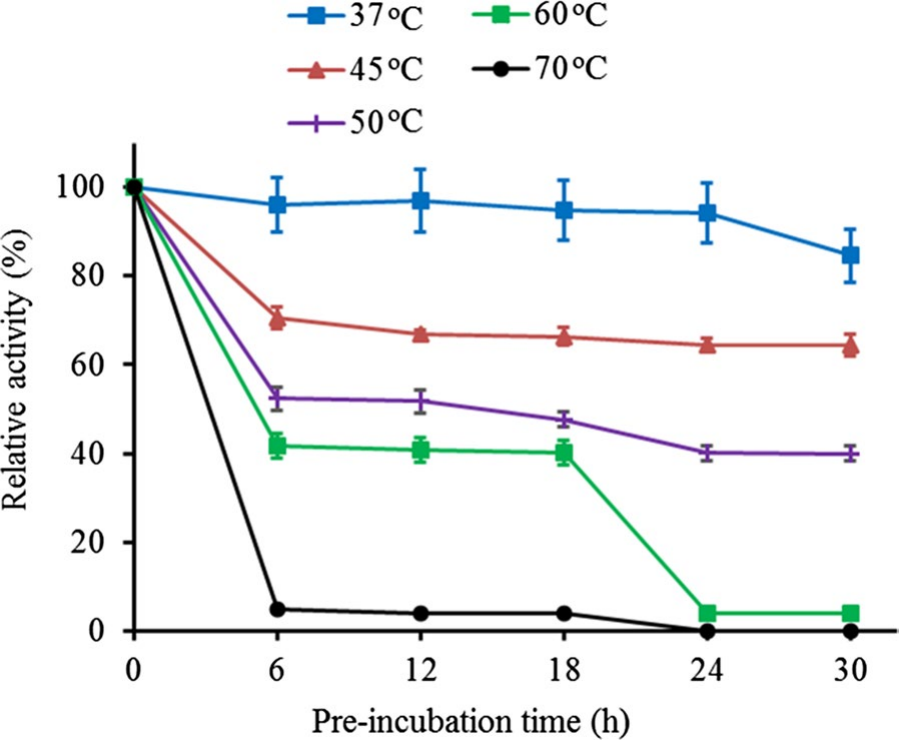
Effect of temperature on the stability of the AMG activity. The enzyme was pre-incubated for 6, 12, 18, 24, and 30 h before the assay was carried out at 45 °C for 1 h (pH 5.0). All error bars are represented as the standard errors of the means (SEM)

The effect of temperature on AMG showed that the enzyme reached its optimum activity at a temperature of 50 °C (Fig. 7). Lowest activities were observed at 4 °C and 70 °C, while no apparent activity was recorded at 80 °C (Fig. 7). The enzyme also displayed its highest stability at 45 °C and was least stable at 70 °C (Fig. 8). The enzyme retained at least 65% of its original activity at a temperature between 37 and 45 °C up to 30 h, while less than 60% activity was retained up to 30 h at a temperature of 50 °C (Fig. 8).

### Effect of metal ions and additives on the activity of AMG

Table 2 shows the effect of the presence of metal ions on AMG activity measured at pH 5.0 and a temperature of 45 °C. Assays were performed with the addition of each metal ion with the concentration ranging from 1 to 10 mM. The chloride salts of these metal ions were used. Manganese (Mn^2+^) and calcium (Ca^2+^) have the highest relative activity values of 112 and 106% at 10 mM concentration. Cobalt (Co^2+^), mercury (Hg^2+^), cadmium (Cd^2+^) and aluminium (Al^3+^) all inhibited the enzyme, with values of 77, 0.45, 4 and 86%, respectively, at the same concentration. Other metal ions tested did not have any significant effect on AMG activity. Table 3, on the other hand, shows the influence of the additives (other chemicals) on AMG activity. SDS had the highest inhibitory effect on the various concentrations used, followed by EDTA at a concentration of 10 mM only, while other tested additives were not inhibitory to the AMG activity.

**Table 2:** Effect of the presence of metal ions on AMG activity

Metal ion	% Relative activity (10 mM)	% Relative activity (5 mM)	% Relative activity (1 mM)
Mg^2+^	98 ± 4.7	99 ± 6.33	100 ± 3.87
Na^+^	102 ± 9.78	100 ± 7.14	101 ± 6.14
K^+^	96 ± 5.66	99 ± 6.74	101 ± 6.97
Al^3+^	86 ± 3.05	95 ± 5.68	100 ± 4.27
Mn^2+^	112 ± 7.41	102 ± 2.8	100 ± 4.98
Ca^2+^	106 ± 5.95	100 ± 2.17	100 ± 2.31
Fe^2+^	98 ± 4.7	100 ± 1.18	100 ± 1.28
Co^2+^	77 ± 3.51	95 ± 2.93	100 ± 3.95
Hg^2+^	0.45 ± 0.05	59 ± 0.19	92 ± 2.23
Cd^2+^	4 ± 0.23	66 ± 2.17	100 ± 3.16

All values are represented by the standard errors of the means (± SEM)

**Table 3:** Effect of the presence of additives on AMG activity

Additive	% Relative activity (10 mM)	% Relative activity (5 mM)	% Relative activity (1 mM)
NaN_3_	100 ± 7.28	102 ± 4.99	101 ± 5.28
NH_4_Cl	101 ± 1.69	101 ± 4.25	101 ± 8.05
EDTA	92 ± 5.59	102 ± 9.5	102 ± 6.73
SDS	5.37 ± 0.32	39.8 ± 1.64	87.9 ± 4.48
Tween20	99.7 ± 1.75	100 ± 4.91	101 ± 5.77
DMSO	99.7 ± 8.12	101 ± 1.45	102 ± 3.12
IAA	99.6 ± 7.69	102 ± 8.88	102 ± 4.83

All values are presented by the standard errors of the means (± SEM)

### Enzyme kinetics

**Fig. 9:**
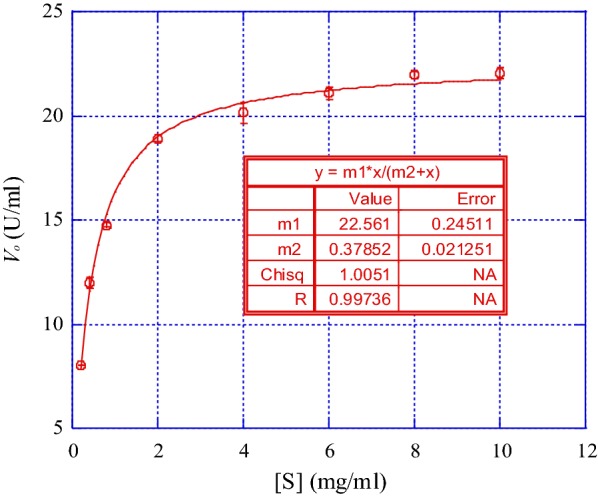
Michaelis–Menten type kinetics of the purified AMG enzyme versus soluble starch concentration. All error bars are represented as the standard errors of the means (SEM) (m^1^ = V_max_ and m^2^ = K_m_)

Figure 9 shows the result of a graph after plotting the concentration of starch [S] versus initial velocity *V*_*o*_ of AMG enzyme activity. [S]/*V*_*o*_ represents the ratio of starch concentration to the initial velocity of the AMG enzyme activity (Fig. 9). *K*_*m*_ represents the Michaelis–Menten constant of the AMG enzyme and *V*_*max*_, the maximum velocity achieved by AMG enzyme-starch reaction. AMG enzyme produced by *L. incrustata* has a *K*_*m*_ value of 0.38 mg mL^−1^ and a *V*_*max*_ value of 22.56 U mL^−1^. Hence, AMG enzyme produced by *L. incrustata* has a high affinity for starch. The turnover number (*k*_*cat*_) and specificity constant were calculated to be 70 s^−1^ and 184 mg mL^−1^ s^−1^, respectively.

## Discussion

Amyloglucosidase enzyme was produced under liquid-state fermentation conditions using a modified Melin–Norkrans medium. The elution profile on Sephadex G-100 was sufficient to obtain a single protein band which confirmed the purity of the enzyme. Thin layer chromatography patterns of the hydrolysis products demonstrated that only glucose was produced as an end product of enzyme activity (Fig. 2). This agrees with the reports that most fungi produced glucose as the product of AMG hydrolysis (Kusuda et al. 2004; Negi and Banerjee 2009; Slivinski et al. 2011). The product of the ammonium sulphate precipitation and the dialysis displayed specific activity values of 15.8 and 21.5 U mg^−1^ protein, respectively (Table 1). The amylase analysis by zymography revealed the formation of a single amylolytic band on the starch-SDS gel (indicative of a single isoform). After gel filtration, the enzyme was found to be homogeneous on SDS-PAGE (Fig. 4) with a specific activity value of 27.174 U mg^−1^ protein at a temperature of 37 °C. Furthermore, the findings on the SDS-PAGE showed many protein bands with no amylase activity except a single band of AMG which had a molecular weight of 101 kDa (Fig. 4). This underpins the reports of some authors that the molecular weights of AMG in most fungi were within a range of 11.5–118 kDa (Hur et al. 2001; Nguyen et al. 2002; Kusuda et al. 2004; Slivinski et al. 2011).

The optimum pH and stability were determined (Figs. 5, 6), the optimum pH for AMG from was pH 4.0, and the enzyme was most stable at a pH of 5.0. A change in the pH of 1.0 unit resulted in decreased activity. The results were similar to the report of Slivinski et al. in 2011, who stated that the pH stability range for *A. niger* was within 4.0–6.0 at temperatures between 40 and 60 °C. Kusuda et al. (2004) also reported that the glucoamylase obtained from an ectomycorrhizal fungus (*L. shimeji*) was most active at around 40 °C (pH 5.0) and stable at pH between 4.5 and 6.5 for 30 min at 37 °C. The AMG from *L. incrustata* was found to exhibit a steady activity at a temperature of 37 °C and 45 °C, retaining over 65% of AMG activity up to 24 h at 45 °C (Fig. 8). The activity declined with increased temperature. The stability of the AMG at 45 °C was an improvement, considering the report of Hur et al. (2001) who revealed that the α-amylase and glucoamylase from *T. matsutake* were only stable at 4 °C to 30 °C for 30 min.

Some metal such as Co^2+^, Hg^2+^, Cd^2+^ and Al^3+^ have been reported to have an inhibitory effect on biomolecules (Tamás et al. 2014). The inhibitory effect of these metals (Co^2+^, Hg^2+^ and Cd^2+^) on the AMG activity was significant to various degrees at all the concentrations tested (Table 2), but more pronounced at a concentration of 10 mM. It has been suggested that inhibition by Hg^2+^ and other heavy metal ions are not only related to binding to the thiol groups but may be the result of interactions with tryptophan residues or the carboxyl groups of amino acids in the enzyme (Selvakumar et al. 1996). For additives (other chemicals), the inhibitory effect of SDS on the AMG activity was the most significant (Table 3), this merely indicated that SDS is a denaturant (Michaux et al. 2008), while other tested additives were not inhibitory to the AMG activity. The AMG kinetics revealed that the activity increased with increased starch concentration, recording a *V*_*max*_ value of 22.56 U mL^−1^ and *K*_*m*_ of 0.38 mg mL^−1^. This corroborates the report of Slivinski et al. (2011) who observed similar results. The AMG from *L. incrustata* had a high substrate affinity which suggested its suitability for industrial applications. Moreso, the AMG turnover number (*k*_*cat*_) was (70 s^−1^) on soluble starch compared well with those of *Humicola* and *Aspergillus* species. Riaz et al. (2012) reported a *k*_*cat*_ value of 69 s^−1^ for *Humicola* sp. and also, a much higher *k*_*cat*_ value (343 s^−1^) was reported for *A. niger* (Riaz et al. 2012). The *K*_*m*_ and *k*_*cat*_ values reported in this study were better than those reported by some authors. For example, 4.5 g L^−1^ and 45 min^−1^ were reported for *Tetracladium* sp. (Carrasco et al. 2017); 3.8 mg/mL and 41.7 s^−1^ for *Paecilomyces variotii* (Michelin et al. 2008) (Table 4).

**Table 4:** Comparison of the kinetic parameters of *Leohumicola incrustata* and other fungi

Organism	*K*_*m*_ (mg mL^−1^)	*k*_*cat*_ (s^−1^)	References
*Leohumicola incrustata*	0.38	70	Current study
*Humicola* sp.	0.26	69	Riaz et al. (2012)
*Aspergillus niger*	0.25	343	Riaz et al. (2012)
*Tetracladium* sp.	4.5	0.75	Carrasco et al. (2017)
*Paecilomyces variotii*	3.8	41.7	Michelin et al. (2008)

In conclusion, the approach of sourcing enzymes from the environment is proving to be useful in producing novel enzymes that are suitable for the bio-economy. Our *L. incrustata* is just one out of many ericoid mycorrhizal root endophytes that could be employed for the production of enzymes for the bio-economy. This present study described, for the first time, the purification and characterization of an amyloglucosidase from *L. incrustata*. Keeping in mind, all the promising characteristics such as temperature stability over an extended period, high substrate affinity, stability to a range of chemicals and being non-pathogenic make AMG a possible candidate for future application in industrial processes, for example, can be used in the production of bioethanol and glucose syrup.

## Abbreviations

AMG: amyloglucosidase
ERM: ericoid mycorrhizal
SDS–PAGE: sodium sulphate dodecyl sulphate–polyacrylamide gel electrophoresis
TLC: thin layer chromatography
PDA: potato dextrose agar
DNS: dinitrosalicyclic acid
BSA: bovine serum albumin
TEMED: tetramethylethylenediamine
DMSO: dimethyl sulphoxide
EDTA: ethylenediaminetetraacetic acid
IAA: indole-3-acetic acid
SEM: standard errors of the means
